# Quasi-3D Modeling and Efficient Simulation of Laminar Flows in Microfluidic Devices

**DOI:** 10.3390/s16101639

**Published:** 2016-10-03

**Authors:** Md. Zahurul Islam, Ying Yin Tsui

**Affiliations:** Department of Electrical and Computer Engineering, University of Alberta, Edmonton, AB T6G 1H9, Canada; mzislam@ualberta.ca

**Keywords:** microfluidics, microfluidic flow simulation, microfluidic cytometer, computational microfluid dynamics

## Abstract

A quasi-3D model has been developed to simulate the flow in planar microfluidic systems with low Reynolds numbers. The model was developed by decomposing the flow profile along the height of a microfluidic system into a Fourier series. It was validated against the analytical solution for flow in a straight rectangular channel and the full 3D numerical COMSOL Navier-Stokes solver for flow in a T-channel. Comparable accuracy to the full 3D numerical solution was achieved by using only three Fourier terms with a significant decrease in computation time. The quasi-3D model was used to model flows in a micro-flow cytometer chip on a desktop computer and good agreement between the simulation and the experimental results was found.

## 1. Introduction

The development of microfluidic devices fabricated using microfabrication technologies began several decades ago [1]. Lab-on-a-chip (LOC) devices and micro-total-analysis systems (*μ*TAS) are still of great interest due to the increasing number of potential applications identified in diverse areas such as chemistry, biology, environment, and biomedical diagnosis and analysis [2,3,4,5,6,7,8,9,10,11,12,13,14,15]. Within the last ten years, a significant increase in research activities in these devices has taken place and microfluidics remains a hot research topic.

A typical *μ*TAS or LOC device consists of a network of micron-sized fluid channels that carry the samples to be analyzed. A theoretical understanding of microfluidic flow is essential for predicting the behavior of flows inside microfluidic chips and to optimize the chip design. Most work on microfluidic systems incorporates fluid mechanics modeling as a design tool or as a way to interpret experimental results. However, there are very few cases in which the geometries are simple enough that analytical expressions for the velocity fields can be obtained. Even in the case of a straight rectangular channel, the solution can only be expressed as an infinite series of orthogonal functions [16]. Thus, early researchers in the field used simplified geometries and simple analytical models well-known from macroscopic fluid mechanics in their work [17,18,19,20].

There are simplified methods for simulating a chip-scale fluid network. One such method is to use the similarity between the hydraulic resistance in a channel [21] and the ohmic resistance in a branch of an electrical network and to carry out the analysis with an electrical simulation tool such as PSPICE [22,23,24]. In this approach, pressure replaces voltage and volume flow replaces current. The main drawbacks of this technique are that the calculation of resistances is complicated, if not impossible, for general flow structures, and the details of the velocity field as a function of position in the channels is not accounted for.

For complex three-dimensional (3D) flow geometries and systems, numerical techniques including finite difference, finite volume, and finite element methods have been used for many years [25,26,27], and a number of commercial software packages are currently available. However, these packages are designed to handle general and arbitrary boundary conditions and therefore require extensive computing resources and time for solving realistic problems. Due to small computational cell sizes, they are typically used to model the flow in limited regions of a complicated structure but are not well suited for larger scale problems. This restriction may be alleviated by using two-dimensional (2D) simulations, but they ignore important effects, such as drag caused by the upper and lower walls of channels, which may significantly affect the solution.

In this paper, a quasi-3D (Q3D) simulation method is introduced for planar microfluidic chips in which the microchannels are approximately rectangular in cross-section due to the nature of the etching or embossing processes in microfabrication. As will be shown below, a Fourier series decomposition of the velocity profile in the direction normal to the planar layers reduces the 3D flow problem to a limited number of 2D problems which can be solved more rapidly than a single full 3D problem without sacrificing accuracy significantly. This approach has been used in the past [28] for simulations of electromagnetic waves in networks of rectangular waveguides.

## 2. Theoretical Background

For an incompressible liquid of mass density *ρ* in which the only forces are viscous drag and pressure gradients, the equations [29] that determine the local velocity, **V**, are the continuity equation,

(1) ∇.V=0

and the Navier-Stokes equation:

(2) $$\rho \;[\frac{\partial V}{\partial t}+(V.\nabla )V]=\mu \nabla^{2}V-\nabla P$$

where *μ* is the dynamic viscosity and *P* is the scalar pressure. Additional body forces acting on the fluid, such as gravity, are generally negligible in microfluidics, and electric forces may be added to the equations, but are not considered here. The fluid flow is entirely determined by the pressure distribution, the incompressibility constraint Equation (1), and the boundary conditions.

The following simplifying assumptions, commonly used in microfluidic simulations, are made here:

1. The boundary condition at a channel wall is assumed to be the “no-slip” condition, V(wall) = 0. This condition is well established even at the submicron level [30];
2. The flow is quasi-static. Therefore, the time derivative in Equation (2) can be neglected. By this, it is assumed that the fluid velocities are sufficiently low so that any change in the boundary conditions results in an instantaneous rearrangement of the velocity and pressure fields to conform to the new conditions;
3. Low-Reynolds-number flow is dominated by viscous forces. So the term ρ (V.∇)V can be ignored for low Reynolds number; i.e., $\left|\rho \;(V.\nabla )V\right|/\left|\mu \nabla^{2}V\right|\approx \rho \left|V\right|\;L/\mu <<1$ [31]. For aqueous flows in microchannels with scale lengths on the order of 50 μm, velocities only need to be much less than 1 m·s^−1^ for this to be valid. For the majority of the cases of interest, their velocities are significantly less than 1 m·s^−1^. Equation (2) then reduces to the much simpler equation:
  (3) $$\mu \nabla^{2}V=\nabla P$$
4. Finally, there are no free-moving bodies (particles) in the flow that can locally disrupt the velocity profile, a condition commonly assumed in microfluidic simulations containing cells or particles in low concentrations, so that the particle-induced hydro-dynamic disturbances in the flow field can be neglected [32].

Equations (1) and (3) comprise a system of four equations with four unknowns. Despite the simple appearance of these equations, analytical solutions for general channel structures are obtainable in only a few special cases and numerical solutions are generally required. 

**Figure 1:**
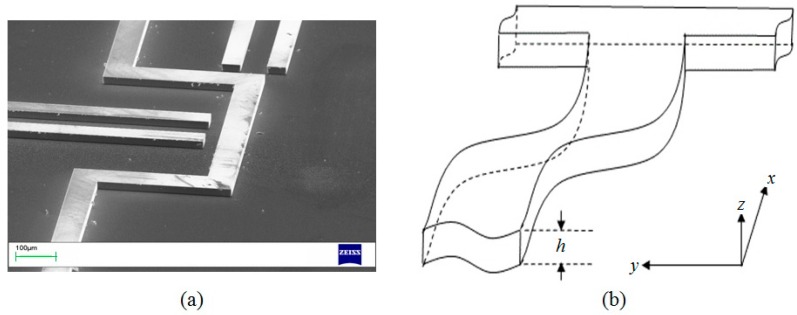
(**a**) Detail of the silicon master formed by reactive ion etching for replicating rectangular microchannels in PDMS; (**b**) Schematic of an *x-y* planar system of rectangular microchannels of height *h*.

In this paper, the analysis is restricted to planar devices in which the microchannels are rectangular in cross-section. Rectangular channels may be formed in glass by deep reactive ion etching (RIE), or by casting polymers such as polydimethylsiloxane (PDMS) on a negative silicon master, also formed by deep RIE. An example of such a silicon master fabricated by our group is shown in Figure 1a. Except for small regions around reservoirs or vias between different levels, the channels are of constant depth, *h*, typically on the order of 50 μm. The coordinate system used here to analyze such a system of microchannels is shown schematically in Figure 1b. The microchannel system is parallel to the horizontal *x-y* plane, and normal to the vertical coordinate, *z*, where 0 < *z* < *h*.

### 2.1. Fourier Expansion of the Flow Field

The velocity field is now expanded in a Fourier sine series,

(4) $$V\left(x,y,z\right)=\sum_{r=1}^{\infty }\left[\left\{\hat{x}\;u_{r}\left(x,y\right)+\hat{y}\;v_{r}\left(x,y\right)+\hat{z}\;w_{r}\left(x,y\right)\right\}\sin\left(r\pi z/h\right)\right]$$

Equation (4) automatically satisfies the no-slip condition, V = 0, at the upper and lower boundaries of the channels. Substitution of Equation (4) into Equation (1) results in,

(5) $$\nabla \cdot V=\sum_{r}\left[\left(\frac{\partial u_{r}}{\partial x}+\frac{\partial v_{r}}{\partial y}\right)\sin\left(r\pi z/h\right)\right]+\sum_{r}\left[(r\pi w_{r}/h)\cos\left(r\pi z/h\right)\right]=0$$

By multiplying Equation (5) by sin(*mπz*/*h*), integrating over the vertical dimension of the device 0 ≤ *z* ≤ *h*, and utilizing the orthogonality property of the sine functions, we have for all indices *m*,

(6) $$\left(\frac{\partial u_{m}}{\partial x}+\frac{\partial v_{m}}{\partial y}\right)=\nabla_{xy}\cdot V_{m}\left(x,y\right)=-\frac{2}{h}\sum_{r}\left(r\pi w_{r}/h\right)\int_{0}^{h}\sin\left(m\pi z/h\right)\cos\left(r\pi z/h\right)dz=\sum_{\begin{array}{l} r\neq m\\ r\pm m=odd \end{array}}\left[\frac{4mr}{h(r^{2}-m^{2})}\right]w_{r}$$

In general, the even Fourier coefficients of the vertical velocity component are coupled to the odd in-plane coefficients and vice versa. However, microchannel flows are dominated by viscosity and in all regions of a device more than a few times *h* away from reservoirs or vias, the vertical components of velocity are negligible and the in-plane flows are symmetric around the mid-plane, *z* = *h*/2 (In Appendix, justification is provided for this statement using full 3D numerical simulations). Thus, away from the inlets with non-zero, vertical input velocities, *w_r_*(*x,y,z*) = 0 for all *r* and *u_r_*(*x,y*) = *v_r_*(*x,y*) = 0 for all even *r*. As a result of Equation (3), $\frac{\partial P}{\partial z}=0$, or P=P(x,y). With these simplifications, the following independent 2D equations for the odd-only Fourier components of velocity are found:

(7) $$\nabla_{xy}^{2}V_{r}\left(x,y\right)-{\left(r\pi /h\right)}^{2}V_{r}={\left(-1\right)}^{\frac{r-1}{2}}\frac{4}{r\pi \mu }\nabla_{xy}P\left(x,y\right)$$

(8) $$\nabla_{xy}\cdot V_{r}\left(x,y\right)=0$$

After solving for the Fourier components of the velocity in the *x-y* plane, the velocity at an arbitrary (*x,y,z*) can be found by summing the Fourier series of Equation (4). The number of terms to be included in the model depends on the accuracy desired and, as will be shown below, this in turn depends on the aspect ratio of the rectangular channels. However, since a much lower number of mesh points can be used than that required for a full numerical 3D solution, the Q3D model is much more time efficient compared to the full 3D numerical solution. In the examples given in the next section, these 2D equations were solved using the commercial finite element method solver, COMSOL.

## 3. Testing of the Model

### 3.1. Straight Rectangular Channel

**Figure 2:**
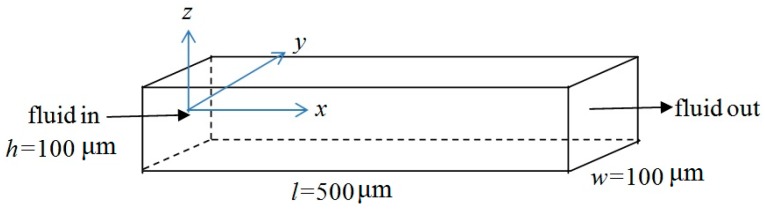
Schematics of the rectangular channel problem used to compare the quasi-3D model with the analytical solution. The midpoint of the channel inlet is taken as the origin of the coordinate system.

To check the accuracy as a function of the number of Fourier terms retained in the model, the Q3D approximation was used to calculate the velocity profiles in a straight rectangular channel of width and height, w and h (flow in the x-direction), and the result was compared with the exact analytical series solution [16]. The first example was a square channel with h=w=100 μm and length = 500 μm. This geometry is schematically shown in Figure 2. At the channel ends, the input and output pressures were set to 100 Pa and 0 Pa, respectively, and Neumann boundary conditions, $\frac{\partial u}{\partial x}=0$, were used for the velocity components. The viscosity of water at 20 °C, μ=0.001002 Pa-s [21], was used in all calculations.

**Figure 3:**
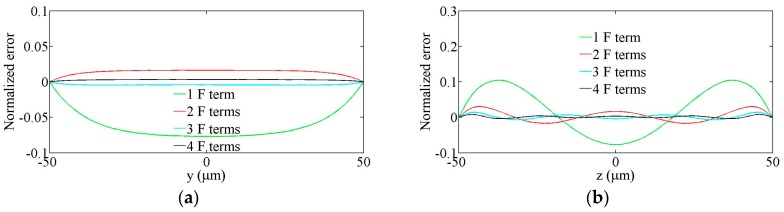
Normalized errors in the Q3D-calculated velocity profiles as a function of Fourier (F) terms in the Q3D model, (**a**) across the horizontal mid-line, *z* = 0; (**b**) across the vertical mid-line, *y* = 0.

At the half-way point in the channel, x=250 μm, the relative errors in the Q3D-calculated values of velocities, *u*, defined as, $\left[u_{A}-u_{Q3D}\right]/u_{A\left(average\right)}$, are calculated (a) across the horizontal mid-line, z=0, as a function of y and (b) across the vertical mid-line, y=0, as a function of z. The results are shown in Figure 3. Note that the relative error was on the order of 1% when only three Fourier terms were used.

**Figure 4:**
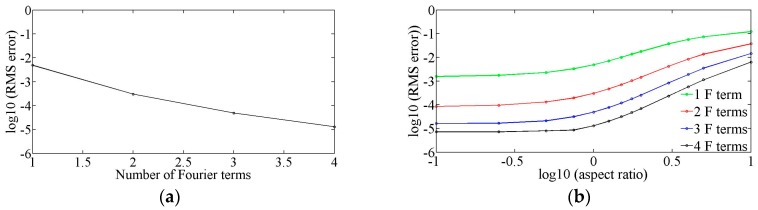
(**a**) Total cross-sectional root-mean-square error as a function of the number of Fourier terms included in the Q3D model for the square channel; (**b**) Aspect ratio, $\alpha =\frac{h}{w}\;$, dependence of the RMS error as a function of the number of Fourier terms included in the Q3D model.

An alternative way of measuring error is the normalized root-mean-square (RMS) error over the whole cross-section defined as $\sqrt{\sum {\left(u_{A}-u_{Q3D}\right)}^{2}/\sum u_{A}{}^{2}}$, where the summations are taken over all analytical ($u_{A}$) and Q3D ($u_{Q3D}$) velocity components and all positions in the cross-section of the channel at *x* = 250 μm. For the square channel example above, the RMS error is less than 1% when only one Fourier term is used, as shown in Figure 4a (in log_10_ units).

The accuracy of a simulation depends on the aspect ratio, α=h/w, of the channel, as shown in Figure 4b. In this figure, the RMSerror (in log_10_ units) is shown for aspect ratios over the range 0.1≤α≤10, and for up to four Fourier terms included in the Q3D model. Here, the width and height of the channel are varied from 10 μm to 100 μm to obtain this range of the aspect ratio of the channel. For wide or shallow channels (log_10_(*α*) < −0.5), the flow profile in the *z*-direction is expected to be nearly parabolic and the RMS error is found to be much less than 1% when only the first Fourier sine term is included in the model. Conversely, for narrow channels (log_10_(*α*) < +0.5), the flow profile in the *z*-direction is expected to be more plug-like and more Fourier components are required for the same accuracy. Still, even with an aspect ratio of 10, the RMS error is less than 1% when only three Fourier terms are included.

### 3.2. Flow in a T-cell

**Figure 5:**
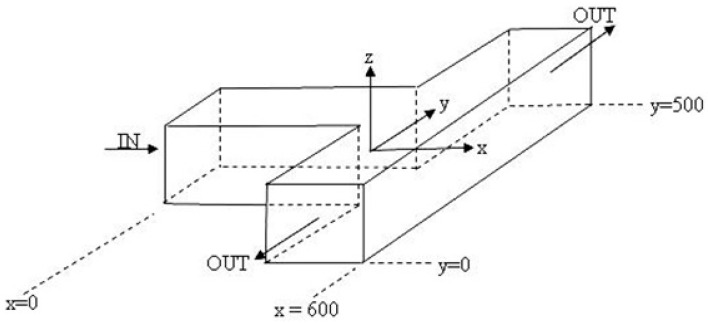
Schematic of the T-cell problem used to compare the Q3D model with the commercial 3D hydrodynamics solver, COMSOL.

**Figure 6:**
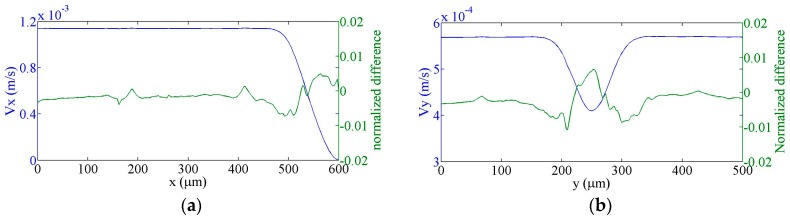
Q3D calculated values of flow velocity and their normalized differences from COMSOL calculations, (**a**) along the centre of the input channel; (**b**) along the centre of the output channels (absolute velocity values).

**Figure 7:**
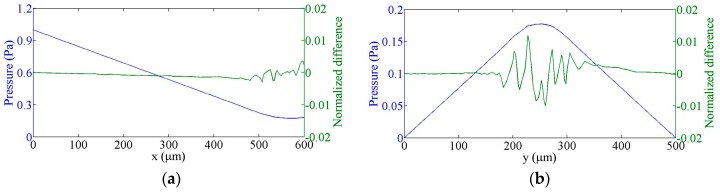
Q3D calculated pressure values and their normalized differences from COMSOL calculations, (**a**) along the centre of the input channel; (**b**) along the centre of the output channels.

The next test of the Q3D approximation is the solution of flow in a T-cell (shown in Figure 5) with three Fourier terms. Since no analytical solution for this system is available, our model results are compared to the results obtained using the full 3D incompressible Navier-Stokes application module of the COMSOL Multiphysics. The input arm is 500 μm long and the output arms are each 200 μm in length and all arms have cross-sectional dimensions of 100 × 100 μm^2^. The x and y velocity components along the centre lines of the input and output arms, respectively, are shown in Figure 6, and similarly the pressure in Figure 7. The differences between the Q3D and COMSOL calculations are indistinguishable on this scale. The normalized differences for either quantity “*q*”, defined as, $\left[q_{3D}-q_{Q3D}\right]/\langle q_{3D}\rangle$, are shown in the same figures with the scale on the right-hand axis. (Here, $\langle q_{3D}\rangle$ is the average value of “*q*” over the channel cross-section.) Note that the normalized difference is less than 1%.

### 3.3. Computation Resources

**Figure 8:**
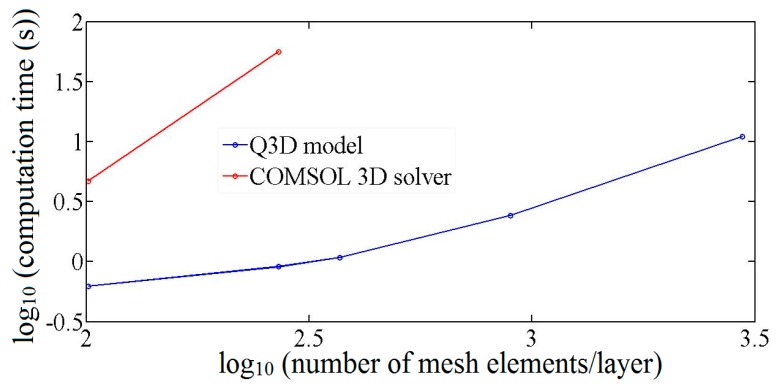
Computation time as a function of the number of mesh elements for the two simulation techniques: Q3D model and COMSOL 3D Incompressible Navier-Stokes module.

The simulations described above were all performed on a desktop PC running COMSOL Version 3.2b. For a direct comparison of computation speed, the same *x-y* mesh pattern was used in the Q3D and COMSOL 3D incompressible Navier-Stokes module, and for the latter, the element pattern was repeated for eight levels vertically. It was not possible to run the COMSOL 3D model with more than 250 elements per level due to higher than available memory requirements. With 250 elements per level, the Q3D calculation was more than 50 times faster than the COMSOL calculation (Figure 8).

## 4. Comparison of Experimental and Simulation Results

**Figure 9:**
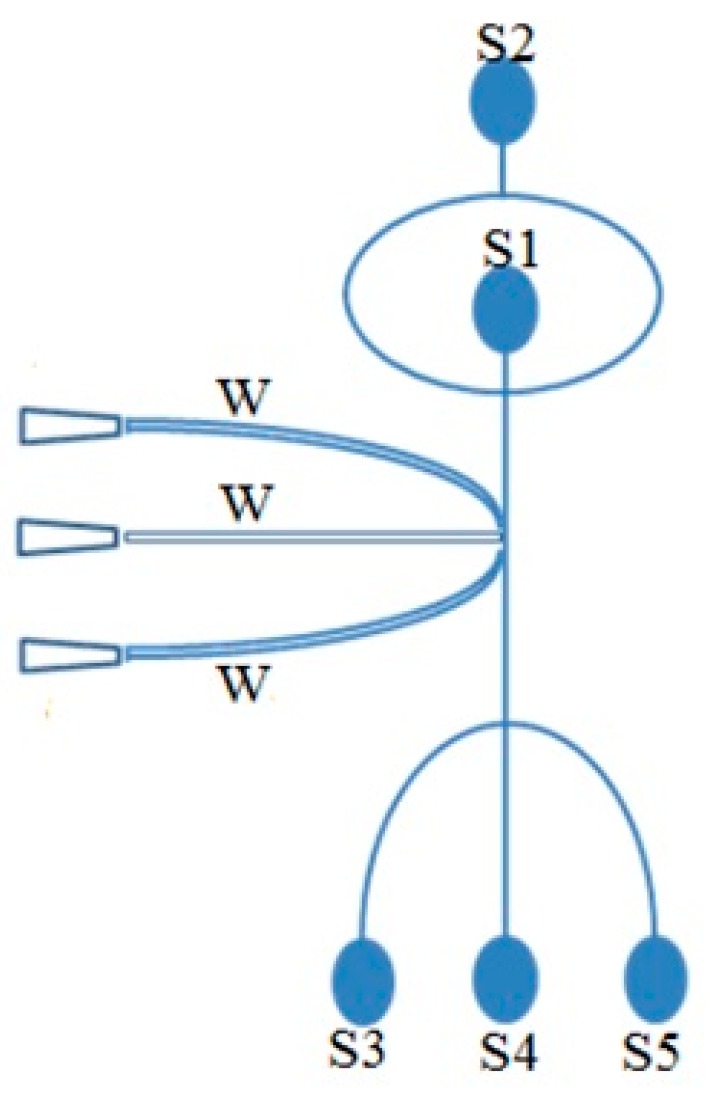
Schematic layout of the microfabricated flow cytometer chip. S1: sample inlet, S2: sheath inlet, S3: side flow switching inlet, S4: default waste outlet, S5: target particle outlet, W: waveguides.

Existing full-3D numerical models typically require extensive computing resources and time for the simulation of chip scale problems. By using our Q3D model, such problems can be simulated on a desktop PC in minutes. As an example, we have simulated the flows in the microchannels of a microfluidic cytometer chip using the Q3D model. One potential application of such a microfluidic cytometer is the sorting of cells from blood [33,34]. The schematic diagram of the cytometer chip is shown in Figure 9. The chip consists of an integrated hydrodynamic focusing system (inlet S2), solid-core optical waveguides (W) and a hydrodynamic side-flow switching system (S3 and S5). The distance from sample inlet S1 to the default outlet S4 is 4 cm and the cross-sections of all the microchannels are 60 μm × 60 μm. A blood cell traveling down the microchannel is optically interrogated, and if it is decided that it should be sorted, a side flow from S3 will be activated, diverting the cell to the outlet S5. A typical micro-flow simulation for the chip requires about 5 min using the Q3D model on the desktop PC as described in Section 3.3. We fabricated the chip and experimentally characterized the flows in the microchannels, allowing us to compare the experimental and simulation results to validate the Q3D model. The fabrication procedure is described elsewhere [34]. The experimental methods used to characterize the flows in the chip and their comparisons to the Q3D simulation results are described below.

### 4.1. Flow Speed Characterization in the Main Microchannel

**Figure 10:**
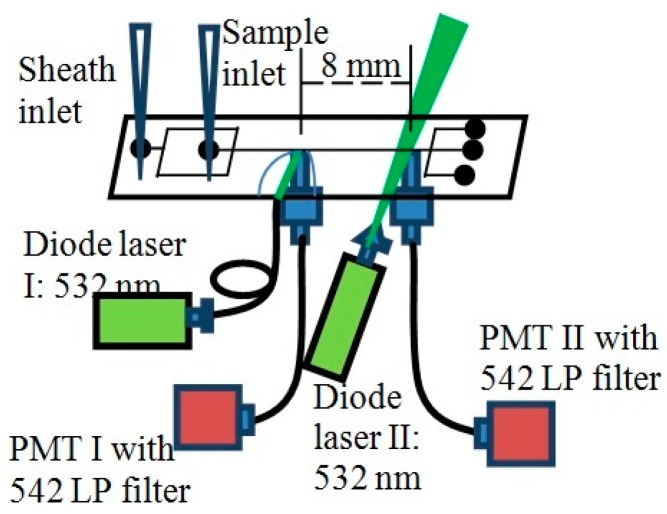
Schematic set-up for the measurement of the transit time of fluorescent particles in the microchannel.

The fluid flow speed inside the main microchannel was estimated by injecting fluorescent plastic microspheres into the chip and measuring their speeds by utilizing the time-of-flight technique. The speed distribution of the microspheres was determined by measuring the transit times of the microspheres between two points of known distance in the channel. A schematic diagram of the experimental set-up is shown in Figure 10. An aqueous solution of the fluorescent microspheres (10 μm diameter, orange fluorescence, FluoSpheres^®^ (Molecular Probes Inc., Carlsbad, CA, USA)) was injected into the chip through the sample inlet. The flow control was accomplished using the MFCS^TM^ system of FLUIGENT [35]. The solution was diluted sufficiently so that on the average, there would be no more than a single microsphere at a time between the inlet and outlet in the main microchannel. The sheath inlet was fed by a de-ionized water source. The particles were interrogated by two 532 nm laser beams at two points of the main microchannel; at the detection region of the chip and at another point downstream, 8 mm apart. The emitted fluorescent signals were collected by two optical fibers (1 mm diameter core) placed under those two regions and were fed to two photomultiplier tubes (PMT) each equipped with a longpass (LP) filter that allowed only light with wavelengths of 542 nm or larger to pass, in order to suppress the 532 nm laser light.

**Figure 11:**
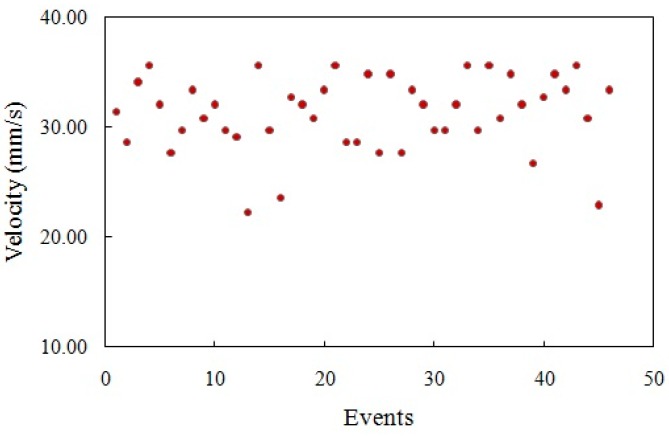
The velocities of the microspheres in the main microchannel for sample and sheath pressure values of 50 and 65 mbar, respectively.

**Figure 12:**
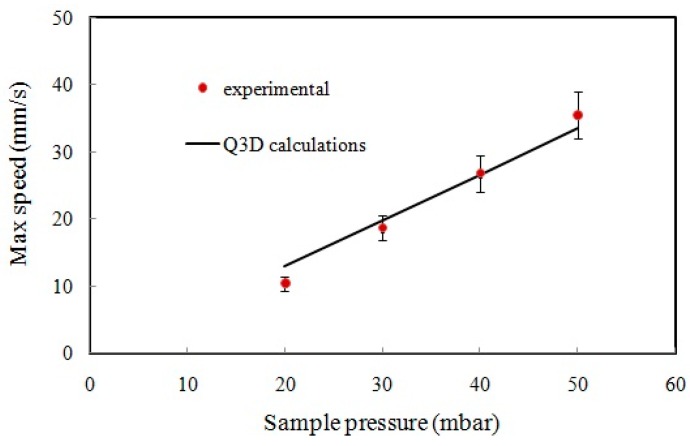
The experimental and Q3D calculated maximum flow speeds at the main microchannel of the chip as a function of the sample pressure.

The velocities of the microspheres obtained from measuring their transit times between the two PMTs in the main microchannel are shown in Figure 11 for sample and sheath pressure values of 50 and 65 mbar, respectively. Most of the particles experienced lateral migration due to the wall effect and the pinching effect in the microchannel flow [36,37]. The particles near the wall of the microchannel were pushed toward the centre due to the wall effect and the particles near the centre were pushed towards the wall due to the pinching effect [36,37]. Only the particles that were flowing through the centre of the microchannel did not migrate laterally due to zero net pinching force at the centre, and these particles moved the fastest, because the flow speed peaked at the centre [37]. Thus, the maximum of the microsphere velocities in Figure 11 can be assumed to represent the flow speed at the center of the channel. The experimental maximum flow speed (speed of the fastest moving particles) and the Q3D calculated maximum flow speed at the main microchannel of the chip as a function of the sample pressure are shown in Figure 12. As the plots show, the experimental measurements agree reasonably well with the values obtained by the Q3D calculations.

### 4.2. Flow Focusing Characterization

**Figure 13:**
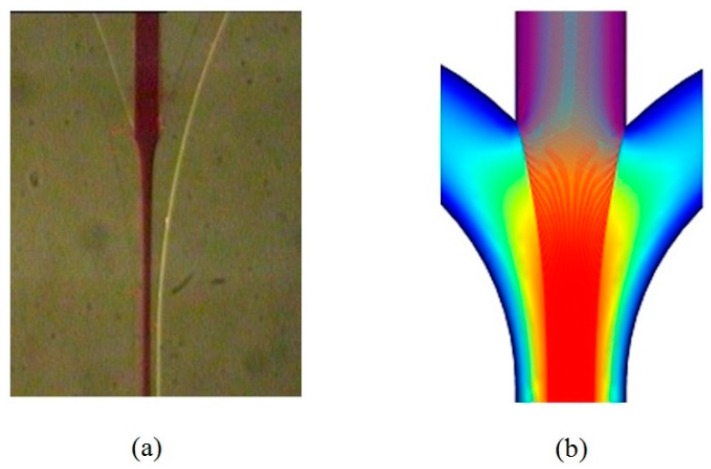
(**a**) A snapshot of the focused flow (experimental); and (**b**) corresponding Q3D calculated flow streams.

The functionality of the hydrodynamic focusing system of the fabricated chip was experimentally characterized by pressurizing liquids of two different colors through the sample (S1) and sheath (S2) inlets and measuring the widths of the focused streams for a range of focusing strength (ratio of sheath pressure to sample pressure) from 0.8 to 1.3. Still images of the flows in the region close to the junction of the sample and sheath channels were captured with a microscope CCD camera image acquisition system (equipped with a 12× magnification lens). The images were taken very close to the junction to reduce the effects of diffusion of the colored fluid into the sheath fluid. A snapshot of the flows with focusing is shown in Figure 13a. The corresponding Q3D calculated flow streams are shown in Figure 13b.

**Figure 14:**
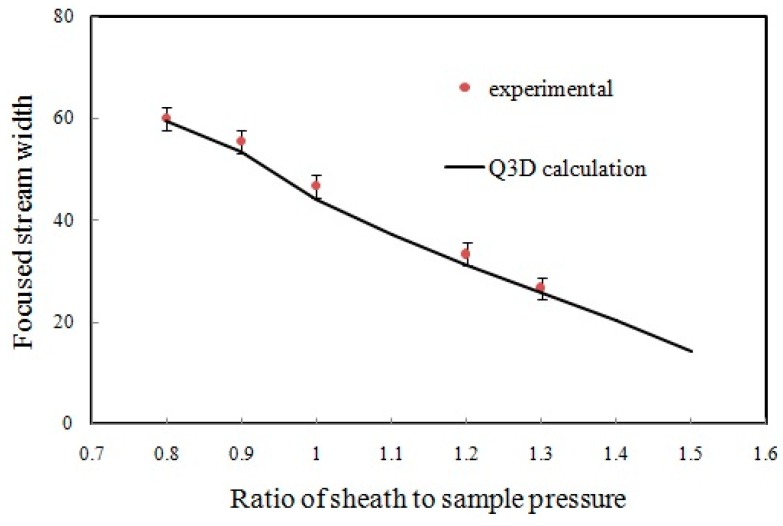
Experimental and simulated focused stream width as a function of focusing strength.

In our experiment, the diffusion of color particles across the boundary results in a color intensity profile across the flow that is not perfectly sharp. To reduce the effects of diffusion in the measurements, we ran this experiment with the highest possible flow rates for the sample and sheath fluids (sample pressure was kept constant at 50 mbar and sheath pressure was varied from 40 to 65 mbar). The width of the focused stream was defined as the full width at half maximum (FWHM) of the color intensity profile of the focused stream across the flow. The experimental values along with those obtained from the Q3D model simulation are plotted in Figure 14. The values from the FWHM estimation lie within 5% of the Q3D numerical predictions.

## 5. Conclusions

In this paper, we have presented a Q3D hydrodynamic flow model for planar microfluidic devices. A Fourier series decomposition of the velocity profile of an incompressible fluid along the height of the microfluidic chip reduced the 3D Navier-Stokes flow equations to several coupled 2D equations. The solution of the Q3D model was compared to that from an analytical model for flow in a straight rectangular channel and to that from the full 3D numerical Navier-Stokes solver for flow in a T-channel. The Q3D model solution was accurate to within 1% of the analytical solution with only three Fourier terms. Comparable accuracy to the full 3D numerical solution was achieved with a significant decrease in computation time. The significant reduction in computation time allows for detailed 3D modeling of microfluidic devices on desktop computers. The Q3D model was used to model flows in a micro-flow cytometer chip on a desktop computer and the simulation results were found to be consistent with the experimental results.

Finally, it should be mentioned that, in spite of the successful applications of the Q3D model and the computational advantages of this model over other numerical approaches, it does have some limitations. The Q3D model is valid only for planar microfluidic devices with geometries and flow parameters such that the flow is characterized by a low Reynolds Number pressure-driven flow. The good news is that most of the microfluidic applications developed that utilize microfabrication technologies fall within these limitations and could be studied by this model.

## Figures and Tables

**Figure A1:**
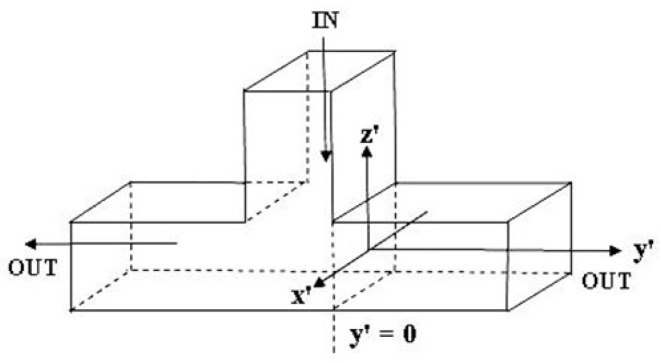
T-cell with a vertical inlet to demonstrate the rapid relaxation to symmetric velocity profiles in the outlet arms.

**Figure A2:**
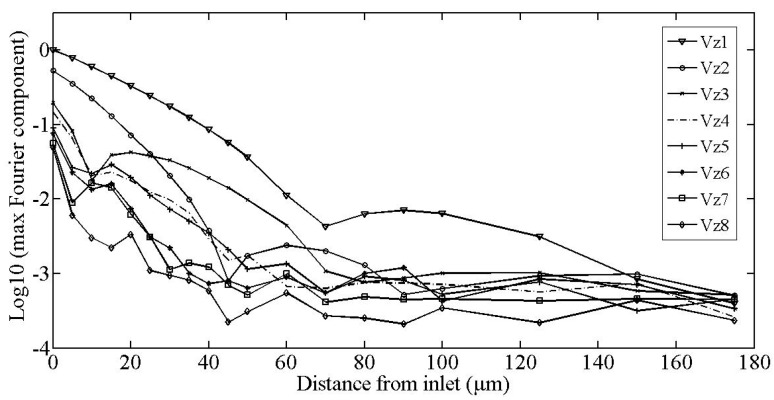
Maximum Fourier components of the vertical velocity components as a function of the distance from the inlet.

**Figure A3:**
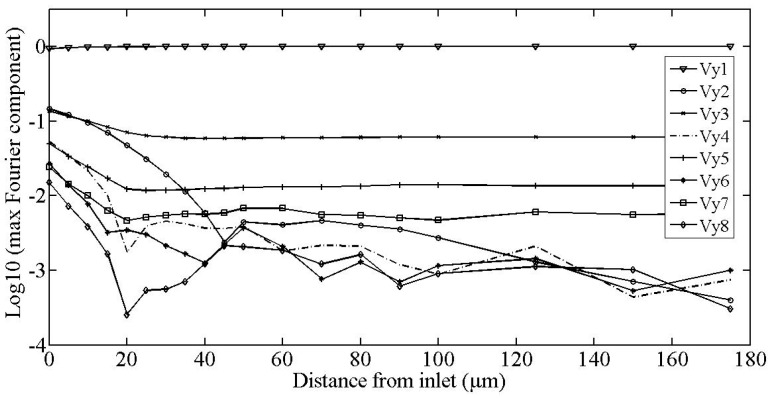
Maximum Fourier components of the axial velocity components as a function of the distance from the inlet.

## Appendix A. Approximation of Symmetric Flow

In Section 2.1, it was stated that short distances away from inlets with vertical flows, the vertical components vanished and the velocity profile could be assumed to be vertically symmetric. To justify this claim, the previous COMSOL 3D results for a T-cell were used except that the input was now considered to be “downward” ($-z^{\prime }$ direction, as shown in Figure A1). The new coordinates $\left(x^{\prime },y^{\prime },z^{\prime }\right)$ now corresponded to (z,y,−x) in the original orientation. The first eight Fourier components of the vertical $\left(z^{\prime }\right)$ and axial output $\left(y^{\prime }\right)$ velocity distributions were calculated at each value of $x^{\prime }$ in the outlet channel up to a distance of 175 μm from the inlet-outlet intersection. The maximum absolute values of these components, normalized by the maximum value at each $y^{\prime }$ position, are plotted in log_10_ units in Figure A2 and Figure A3, respectively. Figure A2 shows that all Fourier components of the vertical velocity component effectively disappeared within 100 μm of the inlet. Figure A3 shows that all even Fourier components of the axial velocity component disappeared, while the odd components also quickly reached the equilibrium values which were expected in a vertically symmetric velocity profile.
